# When does doctor’s recommendation become patient’s resolution? The regulatory potential of procedural justice in the context of compliance with medical advice and the treatment process

**DOI:** 10.3389/fmed.2023.1004994

**Published:** 2023-01-27

**Authors:** Tomasz Prusiński

**Affiliations:** Department of Personality Psychology, Institute of Psychology, The Maria Grzegorzewska University, Warsaw, Poland

**Keywords:** procedural justice, healthcare system, medical advice, patient compliance, doctor’s competence

## Abstract

The main aim of the study was to find empirical evidence to answer the following question: If an important personal interest—namely, health and the prospect of a long life—is not enough to motivate a person to follow recommendations from doctors and medical experts, what factor plays a significant role? The author tested the regulatory potential of procedural justice in this context. The sample consisted of 210 participants, patients of independent primary healthcare facilities and specialist hospital outpatient clinics. The empirical data were analyzed by means of structural equation modeling (SEM). Seven SEM models were tested in the analyses. The adopted analytical strategy brought valuable results. The study supported the main hypothesized relationship, showing that procedural justice was a factor increasing the acceptance of and compliance with medical advice and doctor’s perceived competence. The results of analyses indicate that the fair treatment of patients in healthcare institutions can be regarded as a significant factor regulating patients’ health behavior. The procedural effect is significant for increasing patients’ positive evaluation of doctors’ competence. Also, the evaluation of a doctor as competent increases patient compliance.

## Introduction

Although it seems that compliance with medical advice should be in their best interest, patients often ignore the recommendations received from their doctor during a visit (1). This familiar observation provokes the key question of the present article: If an important personal interest—namely, health and the prospect of a long life—is not enough to motivate a person to follow recommendations from doctors and medical experts, what factor plays a significant role? Despite repeated visits to the doctor’s office, patients continue to choose or adhere to a life style that leads to the aggravation of their disease (2). In these circumstances, a major increase in the cost of healthcare and the presence of avoidable health problems are the main but not the only problems faced by the health service and its beneficiaries. Many solutions have been proposed to address patient non-compliance, but they have not successfully eliminated it.

It seems that the traditional approach, aimed at finding more effective methods of curing diseases, is insufficient. This is because it ignores the entire domain of interactions between the institution or its representatives and the individual who comes for help due to upset somatic and mental balance and due to the lack of resources necessary to restore that balance unaided (3). Nor is health service an area in which legal measures can be applied to motivate people to comply with healthy life guidelines (2). Although legal pressure may intuitively seem to be attractive, its use involves the risk of strong opposition and a decrease in intrinsic motivation to voluntarily follow doctors’ recommendations concerning diseases and preventive healthcare (4). Significant factors regulating compliance with health guidelines should be sought elsewhere. One such factor, whose regulatory potential has not been fully explored, seems to be procedural justice.

### Procedural justice as a regulatory mechanism

The approach assuming that people’s choices are guided by their own interest has dominated many theories explaining human behavior (5). This assumption is so intuitively obvious that, particularly in the domain of health decisions, it has been taken for granted that people are motivated by a desire to achieve better health results. Further empirical research shed a different light on this issue, however.

Contrary to the self-interest assumption, studies in the psychology of justice have shown that people value the way in which they are treated and the quality of the procedures they are required to follow more than they value the outcomes achieved thanks to these procedures (6). They constantly and carefully assess if they are treated fairly (the procedural effect). Fair treatment can therefore be an equally important if not actually a more important factor regulating people’s willingness to cooperate and to choose effective proposals for solving their problems.

Burdziej (7) stresses that it is possible to speak of a significant regulatory potential of procedural justice not only with reference to actions involving authority in a political institution. If the essence of authority lies in the power to impose someone’s will on others (8), a procedural effect is found in every context of institutional functioning. It should also be sought in healthcare institutions, where there is a characteristic dependence of the patient on the physician that involves the decision made, the solution recommended by the physician, and the patient’s resolution regarding compliance with this recommendation (9).

The application of procedural justice as a factor regulating the shape of the final decision makes sense especially from the perspective of the person whom the decision concerns. Naturally, it only makes sense if the person is not in full control of the decision-making process (10)—in situations in which individuals decide to comply or see sense in complying with the decisions of an external arbiter (11). Not having full and direct control over the decision itself, they begin to turn their attention to and evaluate the decision-making process (7). It is this evaluation that determines the acceptance or rejection of the proposed solution and the increase in or loss of trust in the support received from the institution in the future.

### Procedural justice and individual health behaviors: Research findings and gaps

Although hundreds of factors influencing patients’ health behaviors have been identified (12), and probably because multifaceted interventions are considered more effective than those based one factor only (13), the isolated procedural effect is not often explored and requires deeper investigation. Sabate (14) and Prusiński (15) observe that non-compliance with medical advice is generally attributed to the patient’s problem (depression, disturbances of cognitive functions, lack of motivation, rejection, cultural issues, alternative systems of beliefs) without a critical analysis of relational factors. The authors whose research is centered on the quality of relations between the patient and the medical staff focus on the formal and bureaucratic behaviors of medical personnel (16).

As regards research on procedural justice itself, Carroll et al. (17) investigated the regulatory potential of procedural justice as a factor regulating health behavior. They found that older patients who felt they were treated fairly—that is, who described their doctor as respectful and trustworthy—were more often honest with the doctor and more often complied with his or her recommendations concerning treatment. Other studies (9, 18, 19) revealed that patients disliked doctors who used medical jargon, thereby sending signals that they were treating the patient like a child, or ones who treated their patients like objects.

Some of the available data come from meta-analyses concerning factors influencing treatment adherence (20, 21). The conclusions are clear: The quality of patient–doctor communication has the greatest impact on patient compliance. Although, as observed by Tyler et al. (2), good communication is not tantamount to fair treatment, it does impact on patients’ perception of procedural justice. In fact, the opportunity to express one’s opinions and fears, ask questions, and discuss them with one’s doctor is of key significance for perceiving the contact as fair (22). It is possible that, like the quality of communication, also the quality of conduct toward patients during treatment may impact on their willingness to follow medical advice.

In the context of healthcare, it was found that individuals were willing to bear higher costs and suffer health deterioration (in the form of shorter life expectancy) if this was necessary to secure themselves a voice and a chance to participate in health-related decision-making processes (3). Interestingly, previous research (2) suggested that the link between procedural justice and adherence to medical decisions was a cause-and-effect relationship, with procedural justice as the cause. Conducted as an experiment, that research allowed for rejecting the alternative option, with patients’ compliance with doctors’ decisions as the cause that made them more likely to perceive these decisions as just.

Kaptchuk et al. (23) claim that, anchored in relational mechanisms, procedural justice is a potentially valuable explanatory factor. Its presence does not stem from demonstrating to people that following recommendations is good for their health and that it is in their best interest. Being fairly treated by an authority builds a social bond that allows for further identification with and perception of the authority as having the same values and goals (24, 25). As has been established (26), this non-instrumental bond underlies the acceptance of regulations, and their execution is achieved through voluntary self-regulation.

Fair treatment, or procedural justice, is not a homogeneous factor (27). What is the most essential and prototypical for it is the possibility of taking part in the decision-making process by expressing one’s opinion and getting the decision maker’s attention. This component of procedural justice is referred to as voice and has a measurable impact on healthcare decisions (3). Having a voice or what has been called “the voice effect” is one of the central issues people consider when evaluating the reliability of decision-making processes. The refusal of voice leads to the delegitimization of the authority and the institution he or she represents (28). It seems that voice is so psychologically significant in healthcare because it allows the patient to feel important and included, just like the doctor, in the decision-making process (17). This is a powerful motive, whose satisfaction seems to be crucial (29).

Isolating a specific aspect of procedural justice seems insufficient. It is not certain in such cases if other components of the same construct—respect shown to the person whom the decision concerns, neutrality (impartiality) of the authority or a representative of the institution, or trust that the person may have in this representative (30, 31)—also shape health behaviors and whether their contribution strengthens or reduces the regulatory potential of procedural justice as a whole (15).

Procedural justice is relatively often linked with an improvement in the organizational efficiency of hospitals (32) and less often with the development of patients’ health behavior (2). Moreover, procedural justice is rarely measured immediately after the patient’s visit, which makes it difficult to specify what effects of what variables are actually estimated (33).

### The framework of the present study

The aim of the research whose results are presented in this paper was to test the value of procedural justice as a factor increasing compliance with medical advice, willingness to use further medical advice and continue treatment, and satisfaction with the treatment plan and as a factor regulating attitudes toward physicians (i.e., the perception of their competence).

The research also tested if the appraisal of doctor’s competence alone impacted on compliance with medical advice, willingness to use further medical advice and continue treatment, and satisfaction with the treatment plan. To continue the most recent line of research into these issues (3, 17), the author considered several important questions:

Q1: Does the experience of fair procedures in contact with a doctor motivate a person to voluntarily comply with their recommendations?
Q2: Does the experience of fair procedures in contact with a doctor encourage a person to continue to use their medical advice and continue treatment?
Q3: Does the experience of fair procedures in contact with a doctor lead to satisfaction with the treatment plan?
Q4: Does procedural justice play a role in perceiving physicians as competent?
Q5: Does the evaluation of one’s doctor as competent increase compliance with medical advice, willingness to use further medical advice and continue treatment, and satisfaction with the treatment plan?

When exploring these questions, I followed Tyler et al. (2) and Burdziej (7) in assuming that the model of procedural justice, originating in legal institutions, was universal and might also be relevant for patients making decisions about health behaviors. The study was planned to concern people who had experienced what they regarded as fair or unfair interactions with their doctor directly before it and who already had some opinion about the outcomes of their treatment through contact with that particular doctor (33).

The hypotheses were as follows:

H1: The evaluation of contact with a doctor as procedurally just increases the patient’s compliance with the doctor’s recommendations (2, 7, 17, 20).

H2: The evaluation of contact with a doctor as procedurally just increases the patient’s willingness to use further medical advice and continue treatment (2, 17, 20, 21).

H3: The evaluation of contact with a doctor as procedurally just increases the patient’s satisfaction with the treatment plan [(2, 9, 34)].

H4: The evaluation of contact with a doctor as procedurally just increases the patient’s positive evaluation of the doctor’s competence (2, 28).

H5: The evaluation of a doctor as competent increases the patient’s compliance with medical advice, willingness to use further medical advice and continue treatment, and satisfaction with the treatment plan (2, 25).

## Materials and methods

### Participants

The sample consisted of 210 participants: 134 women and 76 men. They were patients of independent primary healthcare facilities and specialist hospital outpatient clinics. Their age ranged from 17 to 79 years (*M* = 42.37, *SD* = 15.49); most of them had secondary (41%) or higher education (38.6%). The sample was balanced in terms of place of residence: 30.5% of the participants lived in villages, 28.6% lived in small towns with a population up to 20,000, and 41% lived in larger towns and big cities in Poland.

### Procedure

The condition for participation in the study was being a patient undergoing treatment for a health problem. Participants in the study were treated for somatic diseases; none of them were psychiatric patients.

Procedural justice was measured directly after the patient had come out of the doctor’s office. Before commencing the measurement, the researcher made sure, by asking a few relevant questions in a conversation, that the patient had just consulted a doctor and that the basic interactions with the doctor during the visit allowed them to form an opinion about procedural fairness and the reliability of the doctor’s work. Patients evaluated treatment for one health problem and the experience of contact with one physician of their choice.

Before the study, each participant was informed about its purpose and asked for consent to take part in it. After consenting, the participant completed the measure—the Procedural Justice Scale—and a survey with questions about sociodemographic data and about variables related to the treatment process. In the present study, I analyzed data collected in a single measurement. Participants received no remuneration. Before conducting the study, I obtained consent from the directors of the healthcare facilities and hospitals where the research was to be held. The study was approved by the Research Ethics Board at the Maria Grzegorzewska University in Warsaw (no decision 209-2019/2020), which raised no ethical objections to the proposed research project.

### Statistical methods

I used the SPSS 26 and IBM SPSS AMOS 26 statistical packages. Preliminary analyses of participants’ sociodemographic data, reliability analyses, and correlation analyses were performed using SPSS 26. To analyze SEM models, I used the AMOS 26 package.

### Measures

#### Procedural justice

In the analyses, procedural justice was treated as an independent variable. To assess it, I used the Procedural Justice Scale (PJS) and its short version, the General Procedural Justice Scale (GPJS; (2, 30)). The full version of this scale (PJS) consists of 14 items (13 positive and 1 negative, no subscales) and yields the patient’s complete, integrated evaluation of the fairness of interpersonal treatment experienced from their doctor. The short version of the scale (GPJS; 2 positive items) operationalizes the general sense of procedural justice. Both versions of the scale are self-report measures, with items to be rated on a five-point scale (1 = *strongly agree* to 5 = *strongly disagree*).

The passage below presents detailed information concerning the quality of the items of both scales. The values of factor loadings (λ) obtained in factorial validity analyses, computed for each PJS item (*I*_1_–*I*_14_), are high or medium: λitem_1_ = 0.86, λitem_2_ = 0.87, λitem_3_ = 0.93, λitem_4_ = 0.84, λitem_5_ = 0.82, λitem_6_ = 0.86, λitem_7_ = 0.90, λitem_8_ = 0.87, λitem_9_ = 0.93, λitem_10_ = 0.86, λitem_11_ = 0.89, λitem_12_ = 0.89, λitem_13_ = 0.89, λitem_14_ = 0.81. High and moderate values were also obtained for the estimated discriminatory power of PJS items: *I*_1_ = 0.86, *I*_2_ = 0.77, *I*_3_ = 0.88, *I*_4_ = 0.77, *I*_5_ = 0.77, *I*_6_ = 0.85, *I*_7_ = 0.83, *I*_8_ = 0.76, *I*_9_ = 0.85, *I*_10_ = 0.82, *I*_11_ = 0.82, *I*_12_ = 0.74, *I*_13_ = 0.76, *I*_14_ = 0.63. The fit statistics from CFA for PJS were as follows: χ^2^/*df* = 2.42, RMSEA = 0.06, GFI = 0.84, CFI = 0.91, TLI = 0.90, SRMR = 0.03.

The values of factor loadings (λ) from the analyses of factorial validity, computed for the two GPJS items (*I*_1_–*I*_2_), are high: λitem_1_ = 0.81, λitem_2_ = 0.84. The values of the estimated discriminatory power of for the two GPJS items were: *I*_1_ = 0.70, *I*_2_ = 0.70. The corresponding fit statistics for GPJS were as follows: χ^2^/*df* = 2.89, RMSEA = 0.05, GFI = 0.94, CFI = 0.96, TLI = 0.95, SRMR = 0.03. The reliability indices of the two scales were α_*PJS*_ = 0.96 and α_*GPJS*_ = 0.92, respectively. Both earlier research (2) and the present assessment of the psychometric properties of the short version of the scale measuring procedural justice show that measurement using this scale is adequate compared to the full version.

#### Demographic variables and treatment context variables survey

The measurement was supplemented with an extensive survey questionnaire that allowed for controlling demographic variables and treatment context variables. The survey included questions about standard sociodemographic data such as sex, age, education, and place of residence; it also included scales assessing dependent variables relevant to the subject of the study: patient’s compliance with medical advice [three items, rated on a five-point scale from *strongly disagree* to *strongly agree*; factor loadings (λ) obtained in factorial validity analyses, computed for the items: λitem_1_ = 0.81, λitem_2_ = 0.85, λitem_3_ = 0.75; discriminatory power: *I*_1_ = 0.63, *I*_2_ = 0.65, *I*_3_ = 0.68], willingness to use further medical advice and continue treatment [three items, rated on a five-point scale from *very unwilling* to *very willing*; factor loadings (λ) from factorial validity analyses, computed for the items: λitem_1_ = 0.85, λitem_2_ = 0.81, λitem_3_ = 0.74; discriminatory power: *I*_1_ = 0.69, *I*_2_ = 0.68, *I*_3_ = 0.64], satisfaction with the treatment plan [four items, rated on a five-point scale from *strongly disagree* to *strongly agree*; factor loadings (λ) from factorial validity analysis, computed for the items: λitem_1_ = 0.86, λitem_2_ = 0.88, λitem_3_ = 0.89, λitem_4_ = 0.92; discriminatory power: *I*_1_ = 0.81, *I*_2_ = 0.78, *I*_3_ = 0.76, *I*_4_ = 0.81], and doctor’s perceived competence [four items, rated on a five-point scale from *strongly disagree* to *strongly agree*; factor loadings (λ) from factorial validity analysis, computed for the items: λitem_1_ = 0.92, λitem_2_ = 0.88, λitem_3_ = 0.85, λitem_4_ = 0.66; discriminatory power: *I*_1_ = 0.79, *I*_2_ = 0.70, *I*_3_ = 0.70, *I*_4_ = 0.60].

I calculated descriptive statistics for the main constructs analyzed in the study, determined the reliability values for the four scales, and computed the correlations between the variables. The results are presented in Table 1.

**TABLE 1:** Descriptive statistics for the analyzed variables, reliability values for measurement scales, and correlations between variables.

Variables	*M*	*SD*	α	Correlations				
				**GPJS**	**Compliance with medical advice**	**Willingness to use further medical advice**	**Satisfaction with the treatment plan**	**Doctor’s perceived competence**
**PJS**	52.65	12.34	0.96	0.81	0.63	0.69	0.81	0.82
**GPJS**	7.85	1.57	0.92		0.57	0.56	0.82	0.83
**Compliance with medical advice**	12.38	2.10	0.80			0.43	0.59	0.62
**Willingness to use further medical advice**	10.61	2.91	0.68				0.62	0.65
**Satisfaction with the treatment plan**	14.63	3.13	0.90					0.86
**Doctor’s perceived competence**	15.80	2.87	0.85					

PJS, procedural justice assessed using the full measure of procedural justice; GPJS, procedural justice assessed using the short measure of procedural justice; M, mean; SD, standard deviation; α, internal consistency of the scale assessing a given variable, estimated using Cronbach’s α; Correlations, pearson’s r correlations between the variables.

All correlations presented are significant at p < 0.001.

As shown by the results presented in Table 1, the values of internal consistency for these scales were high and acceptable, which allowed for performing the measurements. The main constructs: procedural justice, compliance with medical advice, willingness to use further medical advice, satisfaction with the treatment plan, and doctor’s perceived competence are significantly and positively intercorrelated. The values of correlations are high or moderate. The results show that the variables operationalizing these constructs are interrelated. The direction of these relationships is consistent with the hypotheses.

## Results

### Preliminary analyses

To test hypotheses H1–H5, concerning the direction and strength of the linear relationships between procedural justice and treatment context variables, I built SEM structural models:

(1) Separate model with 4 latent variables (for H1, H2, and H3).
(2) Separate model with 2 latent variables (for H4).
(3) Separate model with 4 latent variables (for H5).

Additionally, because H1, H2, H3, and H4 concerned the same procedural justice variable, I built a combined (full, comprehensive) structural model with 5 latent variables (joint model for H1, H2, H3, and H4).

To obtain the best estimations of the analyzed relationships between the variables, I applied different ways of measuring the procedural justice variable within the framework of the same structural model defining the relationships between latent variables. The first measurement proposal was for the procedural justice latent variable to be fully operationalized. In this case, I used the full measure proposed by Tyler et al. (2); the full version of the Procedural Justice Scale, PJS). The second measurement proposal was for the procedural justice evaluation entered into the structural model to be derived from the short scale operationalizing this construct (the simplified version—the short form of the measure, GPJS).

This way of performing analyses of SEM structural models, using different estimations of values for the variable, is recommended by Szymańska (35). Such analyses are feasible if the researcher has more than one scale to measure the variable, as was the case in the present study. This is a valuable procedure that allows for obtaining strong evidence from analyses, because the results from one model can be supported or modified by the results from another model, based on a different operationalization of the main investigated variable.

In total, I tested seven SEM models in the preliminary analyses. Before analyzing the estimation results yielded by structural equation modeling (SEM), I assessed the fit of the structural models with the latent variables (36). The values of fit indices for the measurement models are presented in Table 2.

**TABLE 2:** Fit indices of the tested SEM models.

Hypothesis	Model	χ^2^	*df*	χ^2^*/df*	RMSEA	GFI	CFI	TLI	ECVI	MECVI	CR
**H1–H3**	Full	451.26	249	1.81*	0.062	0.82	0.37	0.30	2.65	2.71	0.97
	Simplified	117.81	51	2.31*	0.079	0.91	0.96	0.94	0.82	0.84	0.97
**H4**	Full	290.23	134	2.17*	0.075	0.85	0.37	0.28	1.74	1.78	0.98
	Simplified	40.65	8	5.08*	0.140	0.94	0.96	0.92	0.32	0.32	0.98
**H5**		140.72	74	1.90*	0.066	0.90	0.69	0.62	0.97	0.99	0.97
**H1, H2, H3, and H4**	Full	588.79	346	1.70*	0.058	0.80	0.30	0.24	3.39	3.48	0.96
	Simplified	236.47	100	2.37*	0.080	0.87	0.94	0.93	1.48	1.51	0.96

Full model = the model with a measurement structure including the results of procedural justice measurement from the full measure of procedural justice (PJS).

Simplified model = the model with a measurement structure including the results of procedural justice measurement from the short measure of procedural justice (GPJS).

χ^2^ = chi^2^ model fit statistic; df = degrees of freedom; χ^2^/df = chi^2^ statistic divided by degrees of freedom; RMSEA = root mean square error of approximation; GFI = index of variance explained by the path model; CFI = comparative fit index; TLI = Tucker–Lewis index; ECVI and MECVI = information criteria for comparing the quality of models; CR = Jöreskog’s composite reliability coefficient.

*p < 0.001.

Using the established criteria for assessing the fit of a theoretical model with a measurement model (35) for SEM models (χ^2^/*df* < 2.5, RMSEA ≤ 0.80; GFI and CFI—values close to or higher than 0.90; TLI—values close to 0.95, ECVI and MECVI—the best model is considered to be the one for which the values of these tests are the lowest), analyzing Jöreskog’s composite reliability (CR) coefficient, and determining the values of path parameters and variance estimating the model, I found that that the simplified structural model, potentially verifying H4, showed poor fit (RMSEA = 0.14, χ^2^/*df* = 5.08). It was not considered in further analyses.

As shown by the values of the most important model fit estimator, RMSEA, the remaining models demonstrate good (full models) or barely acceptable fit (simplified models). The situation is similar when the χ^2^/*df* index is considered; the values of this estimator are lower in the case of full models. By contrast, the values of GFI, CFI, and TLI indices as well as the informative ECVI and MECVI indices are better in the case of simplified models. The reliability coefficients of the constructed models of relations (Jöreskog’s CR values) are similar and high (CR > 0.95).

In view of the above, I decided to consider all the remaining models when testing the hypotheses. The structural models I built are not alternative (competing) for one another, in the sense of being exclusive. I expected that they would support one another on the relationships tested. Thus, further assessment of the hypotheses, based on results from multiple models, allowed for testing the hypotheses with strong empirical support provided.

### Main analyses: SEM results

Hypotheses H1, H2, H3, H4, and H5—postulating links between procedural justice and doctor’s perceived competence as explanatory variables and the explained variables: patients’ compliance with medical advice, willingness to use further medical advice and continue treatment, satisfaction with the treatment plan, and doctor’s perceived competence— were tested using SEM. SEM results are presented in Table 3.

**TABLE 3:** Estimator values for the tested models.

Hypothesis	Model		β	*R* ^2^	*M*β	*MR* ^2^
**H1**	Full	PJ → compliance with medical advice	0.862	0.743	0.786	0.624
	Simplified		0.710	0.504		
**H2**	Full	PJ → willingness to use further medical advice	0.926	0.857	0.871	0.761
	Simplified		0.815	0.664		
**H3**	Full	PJ → satisfaction with the treatment plan	0.936	0.876	0.952	0.890
	Simplified		0.969	0.904		
**H4**	Full	PJ → doctor’s perceived competence	0.955	0.912		
**H5**	Doctor’s perceived competence → compliance with medical advice		0.714	0.510		
	Doctor’s perceived competence → willingness to use further medical advice		0.826	0.683		
	Doctor’s perceived competence → satisfaction with the treatment plan		0.982	0.964		
**H1, H2, H3, and H4 (jointly)**	Full	PJ → compliance with medical advice	0.842	0.709	0.778	0.609
	Simplified		0.713	0.508		
	Full	PJ → willingness to use further medical advice	0.919	0.845	0.873	0.764
	Simplified		0.826	0.682		
	Full	PJ → satisfaction with the treatment plan	0.980	0.960	0.978	0.956
	Simplified		0.976	0.952		
	Full	PJ → doctor’s perceived competence	0.989	0.979	0.994	0.989
	Simplified		0.999	0.999		

β = standardized path coefficient; R^2^ = multiple correlation coefficient; Mβ and MR^2^ = mean values.

Significance of all solutions at p < 0.001.

The values of factor loadings indicating the effect size between the variables were statistically significant in each of the analyzed models, which means all models can be subject to interpretation.

The study supported the main relationship postulated in H1, H2, H3, and H4, showing that procedural justice was a factor increasing compliance with medical advice and regulating the treatment process and the perception of doctor’s competence.

The actual effect of the procedural justice perceived by the patient on satisfaction with the treatment plan proved to be the strongest (Mβ_SEPARATE SIMPLIFIED AND FULL MODELS_ = 0.95, *M*β_JOINT SIMPLIFIED AND FULL MODELS_ = 0.98) compared to the values indicating the strength of the relationship between procedural justice and willingness to use further medical advice and continue treatment (*M*β_SEPARATE SIMPLIFIED AND FULL MODELS_ = 0.87, *M*β_JOINT SIMPLIFIED AND FULL MODELS_ = 0.87) and the relationship between procedural justice and compliance with medical advice (*M*β_SEPARATE SIMPLIFIED AND FULL MODELS_ = 0.79, *M*β_JOINT SIMPLIFIED AND FULL MODELS_ = 0.78).

Likewise, the values of the multiple correlation coefficient *R*^2^ were the highest for the models that presented satisfaction with the treatment plan as determined by procedural justice. The justice–satisfaction models explain 89% (*MR*^2^_SEPARATE MODELS_ = 0.89) to 96% (*MR*^2^_JOINT MODELS_ = 0.96) of the variance in the explained variable. These values are the highest, compared to the corresponding values for procedural justice–willingness to continue treatment models (*MR*^2^_SEPARATE MODELS_ = 0.76, *MR*^2^_JOINT MODELS_ = 0.76) and for procedural justice–patient compliance models (*MR*^2^_SEPARATE MODELS_ = 0.62, *MR*^2^_JOINT MODELS_ = 0.61).

The relationship between procedural justice and doctor’s perceived competence also proved to be very strong (*M*β_SEPARATE FULL MODEL_ = 0.96 and *M*β_JOINT SIMPLIFIED AND FULL MODELS_ = 0.99). The analyzed models explain 91% (*R*^2^_SEPARATE FULL MODEL_ = 0.91) to 99% (*MR*^2^_JOINT MODELS_ = 0.99) of the variance.

The results of analyses also support hypothesis H5. The strength of the relations of doctor’s competence perceived by the patient to the patient’s compliance with that doctor’s recommendations (β = 0.71), willingness to use further medical advice and continue treatment (β = 0.83), and satisfaction with the treatment plan (β = 0.98) is moderately high or very high. The tested models explain 51% (model: doctor’s perceived competence–compliance with medical advice) to 96% of the variance (model: doctor’s perceived competence–satisfaction with the treatment plan).

**FIGURE 1:**
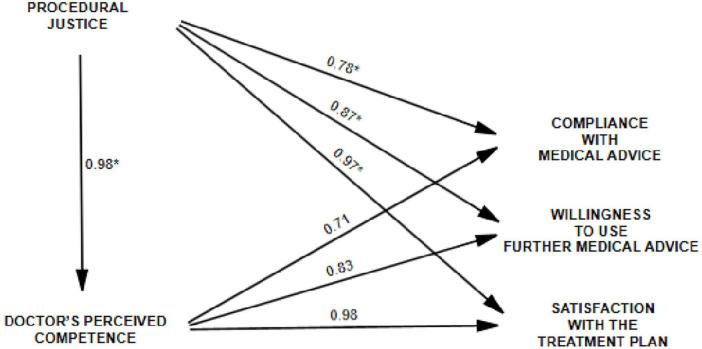
Results of path analysis (standard β path coefficients) for the models of the hypothesized relationships. *Mean value from al the models built and analyzed.

To sum up, Figure 1 presents the paths of relations investigated in the study and the results of analyses based on empirical research in the form of standardized regression coefficients.

## Discussion

Health behavior is basically a domain not covered by legal sanctions or regulations that could motivate individuals to comply with guidelines concerning healthy life and disease prevention. People are usually free to eat whatever they like, do exercise if they want to, or even choose whether to see a doctor and do anything about their health problem or stay at home. There is, of course, a certain regulated area within this domain. People are not allowed to buy any medicines they want or to treat themselves or be treated as they see fit (2). Nevertheless, health is perceived as an aspect of personal life and a sphere of free choice, protected against external regulations.

Numerous studies show, however, that the lifestyle people choose and their health behaviors often lead to diseases and prevent health improvement or recovery. The growing cost of maintaining effective health service has become the main factor behind the pressure to regulate the domain of health behavior and thereby bring people’s behaviors closer to optimal health practices. In this paper I have analyzed one of the solutions that researchers see as having significant regulatory potential.

The results of the empirical research reported in this article indicate that the problem discussed above can be solved, or at least alleviated, by maintaining high procedural justice standards in healthcare institutions. Fair treatment, which makes a person feel important and included in decision making and gives them a sense of having an influence on its final outcome, can be regarded as an important determinant of health behavior. The results suggest that patient’s perception of contact with the doctor as fair and respectful increases compliance with health-related recommendations. Fair and respectful treatment was also found to influence willingness to continue consulting the physician and on satisfaction with the treatment plan.

It turns out, moreover, that the procedural effect is significant for increasing the patient’s positive evaluation of doctor’s competence. In its turn, the evaluation of the doctor as competent, just like fair treatment, increases patient compliance with medical advice, the maintenance of regular contact with the doctor responsible for the recovery process, and the belief that the applied treatment procedures are good. Importantly, the results of analyses from all tested models proved to be consistent in terms of the direction and strength of relationship, and the estimations reported in the present study rank among the highest obtained so far in empirical research (17).

The approach based on the regulatory potential of procedural justice has an important advantage, as it does not require introducing any formal measures or legal instruments. The procedural effect is achieved because patient–doctor cooperation is based on building a relational bond (37). This mechanism is similar to the one applied by psychologists and psychotherapists. Bond is a significant factor in corrective experiences and a vehicle of change for the patient because it allows for maintaining a relationship of cooperation between the helper and the person seeking help (38). By building such professional and, above all, fair and respectful bonds with their patients, physicians maximize patients’ willingness to cooperate and to choose effective proposals for solving their health problems.

It is worth noting one more potentially valuable way of looking for solutions to the problem of developing proper health behaviors, consistent with medical advice. Researchers point out that technologies based on artificial intelligence, deep learning, or machine learning can be taken into consideration in the simultaneous critical analysis of many factors potentially influencing health behaviors (39, 40). If, as researchers insist (12), there are numerous factors that have an impact on patients’ health attitudes, and if it is only the inclusion of all these factors in interventions that effectively shapes the desirable health behaviors (13), then the application of these complex technologies in the health service can be helpful, because these technologies make it possible to simultaneously include and determine the significance of multiple factors. Moreover, by simulating human intelligence in machines programmed to think in ways that resemble human thinking and by imitating the patient’s decision-making process, it is possible to define the most effective solution and to create appropriate motivational programs strengthening compliance with guidelines concerning healthy life and prevention. The above does not negate the value of determining the potential of separate factors, because it is important to know which factors significantly contribute to the regulation of health behaviors.

To sum up, research into a fundamental problem of health psychology has shown that procedural justice shapes patients’ attitudes toward compliance with medical advice by inducing non-instrumental identity-based processes. This new relational approach, though not an obvious one, may speed up the development of healthcare policy.

## Limitations

The various limitations of the present study should be mentioned. In future studies the sample size should be increased, so that the empirical support for SEM models can be stronger. Researchers should make sure that people with different characteristics in terms of extraneous variables are adequately represented, so that analyses including the impact of these variables can be performed in the future. The current sample was too small and too heterogeneous to make it possible to distinguish homogeneous subgroups of subjects. This makes it necessary in the future to identify the potential moderators of the analyzed relationships.

## Data availability statement

The raw data supporting the conclusions of this article will be made available by the authors, without undue reservation.

## Ethics statement

The studies involving human participants were reviewed and approved by the Research Ethics Board at the Maria Grzegorzewska University (APS) in Warsaw, no decision 209-2019/2020. The patients/participants provided their written informed consent to participate in this study.

## Author contributions

The author confirms being the sole contributor of this work and has approved it for publication.
